# The Effect of Long Working Hours and Overtime on Occupational Health: A Meta-Analysis of Evidence from 1998 to 2018

**DOI:** 10.3390/ijerph16122102

**Published:** 2019-06-13

**Authors:** Kapo Wong, Alan H. S. Chan, S. C. Ngan

**Affiliations:** Department of Systems Engineering and Engineering Management, City University of Hong Kong, Hong Kong, China; alan.chan@cityu.edu.hk (A.H.S.C.); scngan@cityu.edu.hk (S.C.N.)

**Keywords:** physiological health, mental health, sleep disturbance, occupational injury, health behaviours, working class, occupational health

## Abstract

There has been no subsequent meta-analysis examining the effects of long working hours on health or occupational health since 1997. Therefore, this paper aims to conduct a meta-analysis covering studies after 1997 for a comparison. A total of 243 published records were extracted from electronic databases. The effects were measured by five conditions, namely, physiological health (PH), mental health (MH), health behaviours (HB), related health (RH), and nonspecified health (NH). The overall odds ratio between long working hours and occupational health was 1.245 (95% confidence interval (CI): 1.195–1.298). The condition of related health constituted the highest odds ratio value (1.465, 95% CI: 1.332–1.611). The potential moderators were study method, cut-point for long weekly working hours, and country of origin. Long working hours were shown to adversely affect the occupational health of workers. The management on safeguarding the occupational health of workers working long hours should be reinforced.

## 1. Introduction

The study of factors affecting occupational health is important to safeguard employees through control of occupational diseases and accidents and to eliminate hazards that threaten the health of workers. Such studies are necessary in order to maintain the quality of work and working environments and to develop a society which ultimately achieves sustainable development [1]. Long working hours are a ubiquitous phenomenon amongst most organisations and companies where the length of time spending on work, comprising main tasks of job, related tasks, commuting, and travel, is too long and detrimental to the health of workers directly or indirectly [2]. Epidemiological studies have shown the negative effects of long working hours on the risks of cardiovascular diseases [3,4,5,6]; chronic fatigue, stress [7]; depressive state, anxiety, sleep quality, all-cause mortality, alcohol use and smoking [3]; and self-perceived health, mental health status, hypertension, and health behaviours [8]. Similar results have been found for long working hours by other studies, for instance, myocardial infarction [9], poor physical health and injuries [10], alcohol consumption, smoking, physical inactivity [11], and depression [12].

### 1.1. Cardiovascular and Cerebrovascular Diseases

Many studies have investigated the effects of different working hours on the occurrence of cardiovascular and cerebrovascular diseases [4,9,13,14,15,16,17,18,19]. A U-shaped relationship between the risk of suffering from myocardial infarction and working hours for Japanese workers was found [17]. Those working less than 7 h per day or more than 11 h per day were at greater risk of experiencing myocardial infarction than with those working 7 to 11 h. Researchers also found that workers in Europe, Japan, Korea, and China who work more than 50 h per week had an increased risk of cerebrocardiovascular diseases [13,17], myocardial infarction [4,9,15], and coronary heart disease [16,19]. However, some findings differ from such results in that working more than 50 h per week decreased the risk of ischemic heart diseases [20] and myocardial infarction [9]. A meta-analysis conducted by Kang et al. [21] indicated that the odds ratio of the effect of long working hours on cardiovascular diseases was 1.37. Another meta-analysis conducted by Virtanen et al. [19] had reported the effect of long working hours on coronary heart disease with a relative risk of 1.39. In the meta-analysis of Kivimäki et al. [22], the risk ratios of the effect of long working hours on coronary heart disease and stroke were 1.13 and 1.33, respectively. The results for working hours and cardiovascular and cerebrovascular diseases are not entirely agreed yet.

### 1.2. Hypertension

The relationship between the chance of suffering from high blood pressure and the duration of working hours has been studied [8,23,24,25,26,27]. Working more than 61 h per week [23,24] showed an increased risk of suffering from elevated systolic blood pressure. By contrast, a few studies suggest that there is a decreased risk of hypertension for those working more than 8 h per day [24] or working 60 h or more per week [25]. Furthermore, Tarumi et al. [26] demonstrated that there is no linkage between circulatory system diseases, hypertension and long working hours. The results regarding risk of experiencing hypertension as a result of long working hours are not consistent.

### 1.3. Diabetes Mellitus

Diabetes mellitus is one of the diseases shown to be related to long working hours. It is associated with daily diet and long working hours, and long working hours may cause workers to the change their eating habits [28,29]. However, some studies have suggested a negative relationship between diabetes mellitus and working hours [29]. The relationship between overtime working and diabetes mellitus is not very straightforward.

### 1.4. Depression and Anxiety

Several studies have found an association between depression and long working hours [12,30,31,32,33,34]. Working more than 34 h per week [33], 55 h per week [34], and 48 h per week [30] increased the chance of experiencing depression and anxiety. A recent study by Ogawa et al. [32] investigated the effects of long working hours on depression symptoms for Japanese residents and found that compared with the residents working less than 60 h per week, those working 80 to 99.9 h per week and more than 99.9 h per week had a 2.83 and 6.96, respectively, greater risk of experiencing depression. By contrast, other studies have found that working 41 to 55 h per week [34] and 41 to 52 h per week [12] was associated with a decreased risk of suffering from depression and anxiety compared with those working less than 41 h per week. Further, it has been reported that female workers have a higher risk of experiencing depression and anxiety than male workers when working the same number of hours [33]. Apart from when working long hours, the results about the effect of long work hours to depression and anxiety are not entirely clear.

### 1.5. Work Stress

Some studies have demonstrated that long work hours contribute to psychological stress and work stress [35,36,37,38]. Working 10 or more hours per day [37], 40 or more overtime hours per month [38], and 60 or more hours per week [36] tended to create stressful feelings. Lee et al. [36] found that working more than 45 h per week decreased the risk of psychological stress. The relationship between working long hours and work stress requires more investigation.

### 1.6. Health Behaviours

Health behaviours, for instance, smoking, alcohol consumption and physical inactivity, are associated with long working hours [33,39,40,41,42]. Shield [33] investigated male and female working populations working more than 34 h per week in Canada from 1994 to 1997. The findings were that increased rates of smoking, drinking and physical inactivity for male workers were 9%, 34% and 43%, respectively, during the period studied, and the increased rates of smoking, drinking and physical inactivity for female workers were 7%, 25% and 41%, respectively. However, Park et al. [41] indicated that there was no difference in smoking amongst three groups of engineers in term of their working hours, ranging from less than 60 h per week, 60 to 70 h per week, and more than 70 h per week. Some studies have reported a significant decrease in physical activity for workers on overtime [41,42]. Further, it has been reported that long working hours are not related to physical inactivity [33,39]. The literature has not reported consistent findings on the relationship between overtime work and health behaviours.

### 1.7. Sleep and Fatigue

Long working hours or overtime work reduce the time for sleep resulting in fatigue [30,39,43]. A normal duration for sleep is about 7 to 8 h per night, which can lower the risk of acute myocardial infarction, cerebrocardiovascular diseases, diabetes mellitus and high blood pressure, as well as reducing working injuries and mistakes [44,45,46]. Furthermore, a significant detrimental effect on quality of sleep is brought about by long working hours [30,47]. Some studies have found that sleep deprivation is directly linked to cardiovascular diseases and high blood pressure [27,48]. Therefore, the duration and quality of sleep of workers may lead to exhaustion and various illnesses. However, by contrast, Bannai and Tamakoshi [3] found that working more than 40 to 60 h per week decreased the risk of problems related to sleep. The results on sleep and working hours are not consistent.

### 1.8. Occupational Injury

Excessive working hours increase the risk of occupational injury. Studies on the effect of long working hours on occupational injury have shown that overtime working increased the risk of occupational injuries [10,49,50,51]. Further, a study found that working 12 or more hours per day and 60 or more hours per week increased the risk of occupational injury [49]. Grosch et al. [10] reported an increase in occupational injuries when working more than 70 h per week compared to those working 41 to 69 h per week.

### 1.9. Aim of the Study

Long working hours have been a controversial issue starting from 1980s, when it was reported that a Japanese design engineer died of brain haemorrhage due to working 2600 h per year [52]. Subsequently, the governments of Japan, Korea and Taiwan recognised that the number of deaths from overwork among workers was increasing. This phenomenon of ‘working to death’ was also reported to have occurred in many organisations and factories in China [53]. Long working hours became a focal issue among Western countries, for examples, the United States, the United Kingdom, Canada, France and Germany [54]. This interest in long working hours resulted in studies to investigate the impact of long working hours on the health of workers in a variety of industries, for various health symptoms, and for ranges of social status [5,19,55]. In general, the findings were that long working hours have adverse effects on health. In spite of the importance of long working hours, apart from the meta-analysis conducted by Sparks and Cooper [5] discovering a slightly positive correlation between various health syndromes and long working hours, there has been no subsequent meta-analysis examining the effects of long working hours on health or occupational health. The issue of long working hours is of great importance in every society, and therefore, it is necessary to investigate the effects of long working hours on the health of workers to identify any changes in the severity of such effect after the previous study conducted in 1997. Further, few studies have addressed the effects of working class, i.e., collar colour occupations, on the association between long working hours and occupational health. It has been argued that occupation is a factor in health inequality among workers [56,57]. Therefore, the influence of occupation on the association between long working hours and occupational health needs to be investigated.

In this paper, a meta-analysis was conducted to synthesise the data from studies from 1998 to 2018 on the effects of working long hours on the occupational health of employees. The purpose here was to examine the relationship between the length of work hours and the occupational health of workers.

## 2. Methods

### 2.1. Literature Search and Selection

The literature to be selected for the meta-analysis was obtained from several electronic databases by browsing keywords related to long working hours and occupational health for the period 1998 and 2018. The selection of this period was due to the shortage of comprehensive analysis on the effects of working long hours on health prior to the study by Sparks and Cooper [5]. The published research for the analysis of the effects of long working hours on occupational health was collected from Google Scholar and Medline (PubMed) by searching the following keywords: (long work hours OR overtime) AND (occupational health OR heart diseases OR cardiovascular disease OR stroke OR diabetes OR blood pressure OR injuries OR pain OR stress OR depression OR anxiety OR exhaustion OR sleep OR smoke OR alcohol OR physical activity). All published papers extracted for the meta-analysis were in English. The abstracts of the published papers selected were screened, and the references were all manually checked to identify if the studies cited and described in the papers were appropriate for conducting this meta-analysis. A total of 1423 papers were collected for inclusion in this stage.

**Figure 1:**
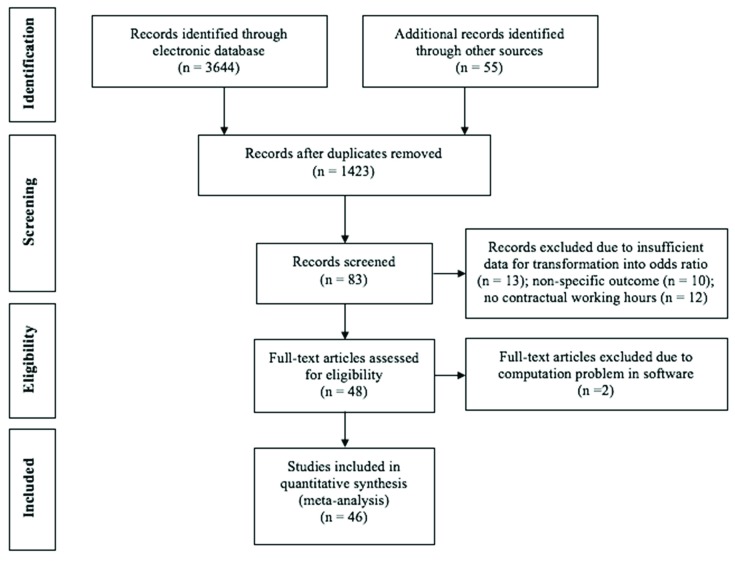
Flow diagram of the study selection process.

Subsequently, the studies were examined to ensure that they fulfilled certain criteria for meta-analysis. First, only studies investigating the daytime workers with the provision of working hours were extracted. Therefore, the studies involving night shift-work schedule and overtime without providing contract hours or regular working hours, for instance, Akerstedt et al. [58] and Sato et al. [38], were excluded from further analysis. Second, papers providing insufficient data for the calculation of odds ratios with 95% confidence interval, such as Shields [33], Beckers et al. [59] and Jeon et al. [60], were excluded. Forty-eight published papers were ultimately selected at this stage. Figure 1 shows the flow diagram of paper extraction for this meta-analysis.

### 2.2. Coding Procedures

The selected studies fulfilling the inclusion criteria were coded into different categories. Regarding the attributes of the participants, four categories were adopted to describe the information extracted, namely, number of participants, country of origin, gender, and working class. The category of number of participants involved the total sample size with, if available, the number of males and females. The category of working class included white collar occupations (management and professional), pink collar occupations (nursing, teaching, and service-oriented work) and blue collar occupations (physical and manual labour workers). The features of the studies were coded into five categories, namely, study design, diagnosis method, reference working hours, different working hours and health measure. Case-control study, cross-section study and prospective cohort study were the main study designs selected, and the follow-up years were extracted for available prospective cohort studies. Self-reports and health or medical examinations were the major diagnosis methods used to evaluate the health conditions of the participants in the studies. The reference working hours were the working hours determined by the authors of a study as the base for an odds ratio of 1.00. The working hours in each selected study were considered for analysing their effects on the occupational health of workers. In the meta-analysis, only the working hours longer than the reference working hours and their corresponding odds ratios were included in the analysis. The category of health measure was to identify occupational health conditions. The occupational health conditions were coded into five categories: (1) physiological health (PH), (2) mental health (MH), (3) health behaviours (HB), (4) related health (RH), and (5) nonspecified health (NH). The classification of the occupational health conditions was based on the meta-analysis conducted by Sparks et al. [5], who explained that the occupational health problems arising from long working hours covered a broad spectrum of health measures and that length of working hours will influence various aspects of health in different ways. Therefore, the health measures were sorted to identify the relationship between different length of working hours and various aspects of health. Physiological health refers to the functions of the body which can be affected by a large variety of diseases and disabilities [61]. Mental health is the subjective recognition of potential competence to cope with stresses in life [1]. Health behaviours involve a number of activities for the prevention of diseases and the enhancement of well-being, including healthy diets, exercises, no smoking and no alcohol drinking [62,63]. Regarding related health, according to Sparks et al. [5], sleep and fatigue are not easily categorised into physiological health or mental health. Hence, related health was defined to cover health measures associated with sleep and fatigue. Further, injury can result from chronic accumulated exhaustion [64], which cannot be categorised as physiological or mental aspects of health [1,65,66]. Therefore, injury and hurt were placed in the category of related health. For situations where health measures were not specified, the category of nonspecified health was applied. The odds ratios with 95% confidence interval between working hours and health measures were calculated to identify relationships between working hours and health measures.

### 2.3. Meta-Analysis

Statistical analysis on the relationships of different lengths of working hours and occupational health conditions (PH, MH, HB, RH and NH) based on odds ratios was used for the meta-analysis. There are two common statistical models in meta-analysis, namely, the fixed effects model and random effects model. The random effects model was adopted in the meta-analysis here due to the variety of effects in the studies caused by different variables, such as study designs, method of data collection and adjustment for the results involved in the studies [67]. The odds ratios produced by the random effects model were obtained from the meta-analysis. The consistency of the results was tested by the heterogeneity indicator, I-squared (I^2^) statistic. The value of I^2^ shows the variations of the studies in term of percentage [68,69]. The greater the value of I^2^, the more considerable the heterogeneity, and a value of zero means homogeneity. Furthermore, the publication bias of the five effect sizes was tested by the trim and fill analysis in which an asymmetry shape in the funnel plots implied the existence of publication bias [70]. The vertical axis with precision was used to detect publication bias in the meta-analysis [71]. Seven variables (gender, diagnosis method, study design, cut-off point for long working hours, working class, country of origin and health measure) were selected to identify possible effects on heterogeneity [72].

## 3. Results

### 3.1. Characteristics of Selected Papers

Forty-eight papers were selected for this meta-analysis before the coding procedures. The odds ratio (OR) for two papers [73,74] could not be generated by the Comprehensive Meta Analysis software (Biostat, Inc., Englewood, NJ, USA) used because log values for lower and upper limits were not symmetric. Therefore, these two records were excluded, and data from the remaining 46 papers with 243 records were used for the meta-analysis. Amongst the 46 papers, 12 were used in a previous meta-analysis conducted by Kang et al. [21], Virtanen et al. [19] and Kivimäki et al. [22,75]. Kang et al. [21] selected the effects with the longest working hours in each target. However, in this paper, the effects of the working hours being longer than the reference working hours in each health-related illness were extracted for conducting the meta-analysis. Virtanen et al. [19] focused on the comparison of the effect sizes in prospective studies and case-control studies. However, this paper examined the effects of a prospective cohort study, case-control study and cross-sectional study. Further, there were two meta-analyses using both published and unpublished data conducted by Kivimäki et al. [22,75]. Kivimäki et al. [75] investigated the influence of socioeconomic status stratification on the overall effect size. Nevertheless, the effect of working class was explored in this paper. Kivimäki et al. [22] assessed effect sizes by categorising the weekly working hours into five groups. However, a cut-off point for weekly working hours and daily working hours was adopted to evaluate the effects. The results of the meta-analysis reported here are shown in Table S1, which gives information and odds ratios for each record based on the coding procedures criteria. Table 1 summarises the percentage contributions of the characteristics of each of the selected 46 papers to this analysis. As can be seen, 26.09% of the papers were published between 1998 and 2007 and 73.91% between 2008 and 1998. For study design, the percentages of case-control study, cross-sectional stud, and prospective cohort study were 10.87%, 54.35% and 34.78%, respectively. There were 61.59% of studies in Asian countries, namely: Japan (36.59%), Korea (19.57%) and China (5.43%). The remaining 38.41% studies were Western countries, namely: the United Kingdom (15.94%), Spain (4.35%), the United States (6.52%), Finland (0.72%), Australia (1.09%), Denmark (4.35%), Sweden (2.17%), Italy (2.17%) and New Zealand (1.09%). The percentages of the number of males and females in the studies were 58.73% and 41.27%, respectively. The total sample size for the meta-analysis was 814,084 participants. Of the 46 papers, the study of O’ Reilly and Rosato in 2013 [76] contributed the largest number to the sample size (414,949). The study of Fukuoka et al. [9] contributed the smallest number to the sample size (97) in their case-control study.

### 3.2. Random-Effects Model of Long Working Hours and Occupational Health Conditions

The coding procedures for classification of the health measures for the meta-analysis were conducted to compare the five occupational health conditions: physiological health (PH), mental health (MH), health behaviours (HB), related health (RH) and nonspecified health (NH). Table 2 shows the number of records, odds ratios with 95% confidence interval, the heterogeneity of the random-effects model and the adjustment for publication bias for each occupational health condition. The number of records on conditions of physiological health, mental health, health behaviours, related health and nonspecified health were 85, 55, 35, 54 and 14, respectively. The overall odds ratio between long working hours and occupational health conditions was 1.245 (95% CI: 1.195–1.298). Of the five conditions, related health had the highest odds ratio of 1.465 (95% CI: 1.332–1.611), followed in decreasing values by mental health (OR: 1.366; 95% CI: 1.238–1.507), physiological health (OR: 1.177; 95% CI: 1.102–1.257), health behaviours (OR: 1.110; 95% CI: 1.004–1.204), and the smallest odds ratio was nonspecified health (OR: 1.065; 95% CI: 0.942–1.204).

**Figure 2:**
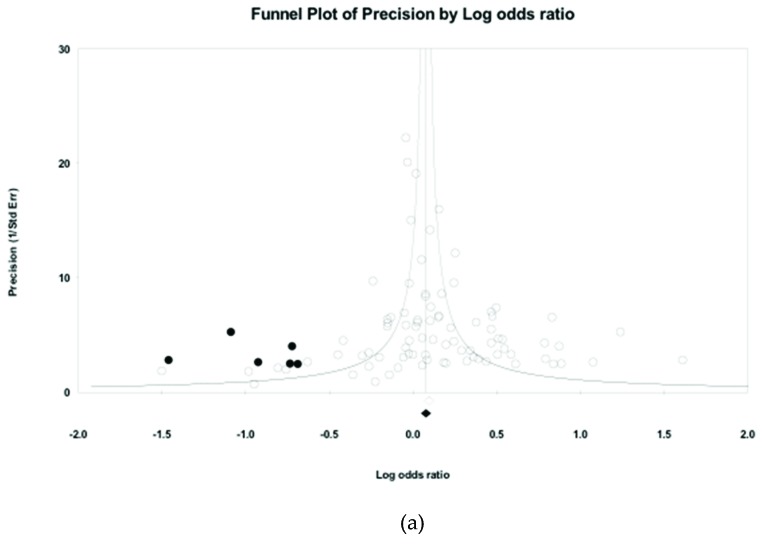

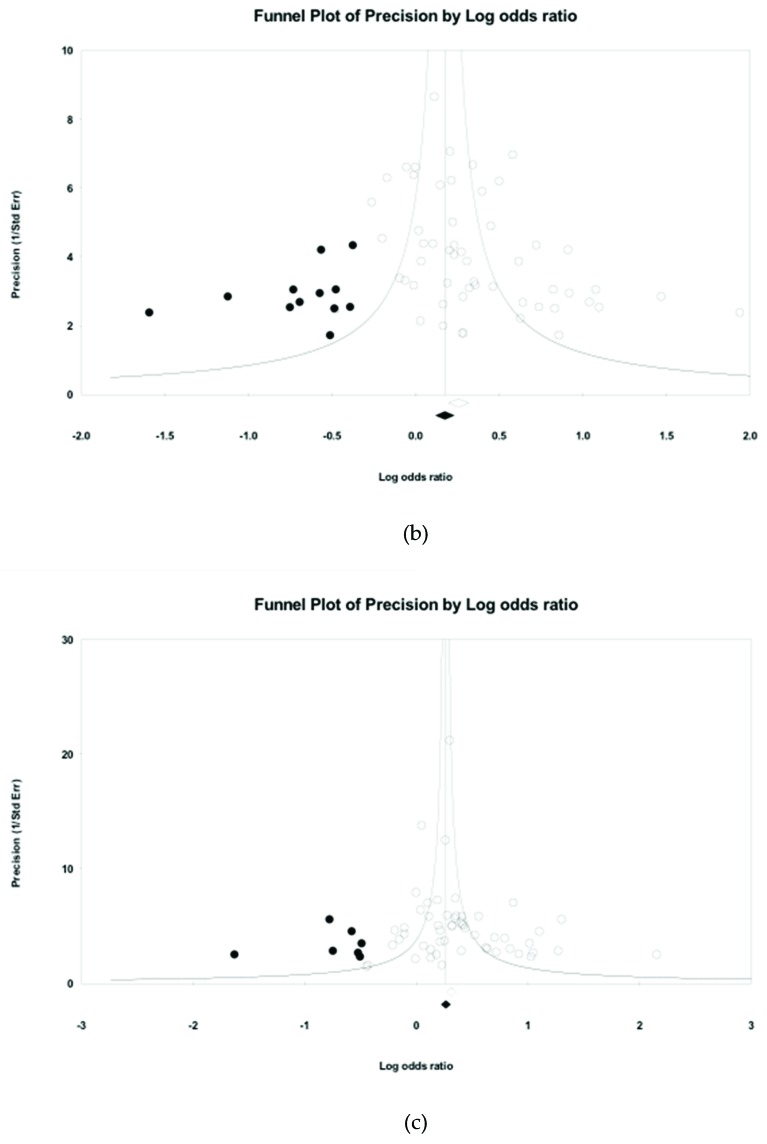
Funnel plot of precision by log odds ratio of long working hours on occupational health. Hollow circles are original data and solid circles are imputed data after adjustment for publication bias. (**a**) Physiological health, (**b**) mental health, (**c**) related health.

The presence of publication bias was assessed by the trim-and-fill analysis. To adjust the publication bias in the meta-analysis, new data points were imputed to the funnel plot to achieve a homogeneous result. Publication bias was found for the conditions of physiological health, mental health and related health (see Table 2). For physiological health, six new data points were imputed in the funnel plot, and the odds ratio decreased to 1.118 (95% CI: 1.041–1.200). Twelve new data points were imputed to the condition of mental health, and the odds ratio decreased to 1.197 (95% CI: 1.072–1.336). There were seven new data points for the related health condition to adjust for the presence of publication bias, which produced a smaller odds ratio of 1.323 (95% CI: 1.188–1.473). The conditions of health behaviours and nonspecified health showed homogeneous results. Figure 2 shows the adjustment for the publication bias for the conditions of physiological health, mental health and related health.

The statistic of I^2^ was used to examine heterogeneity (Table 2). The value of I^2^ for the conditions of physiological health, mental health, health behaviours, related health and nonspecified health were 67.13%, 55.73%, 59.66%, 68.68% and 63.54%, respectively. Considering that 50% represented a substantial heterogeneity [68,69], the heterogeneity was a problem for these five conditions. Therefore, moderator analysis was conducted to identify the potential sources of the heterogeneity.

### 3.3. Moderator Analysis

A moderator analysis was conducted to investigate the possible sources of the heterogeneity using a meta-regression (Table 3). The moderators selected for the meta-regression analysis were gender (only for studies that reported separate effect sizes for males and females), diagnosis method, study design, cut-off point for long working hours (excluding studies that investigated working hours overlapping the cut-off point for long working hours of 50 working hours per week, or 10 working hours per day), working class, country of origin and health measure. The hypothesis for the effect size variations caused by these seven factors was tested by the *p*-values of meta-regression. The meta-regression *p*-values for the gender, study design, cut-off point for long working hours, working class and country of origin, were 0.055, 0.209, 0.000, 0.000, 0.517 and 0.000, respectively. The effects of health measures were grouped in five categories (PH, MH, HB, RH and NH). The condition of nonspecified health was precluded because there was only one subgroup. The meta-regression *p*-values of PH, MH, HB and RH were 0.000, 0.407, 0.521 and 0.048, respectively. The effects of study design, cut-off point for long working hours, country of origin and health measure in the conditions of physiological health and related health were statistically significant. The effect size was not influenced by gender, diagnosis method, working class, mental health or related health.

For study design, the effects were statistically significant for case-control study and cross-sectional study (*p* < 0.001), with odds ratios of 1.811 and 1.338, respectively. The impact of long working hours on occupational health was stronger for case-control study than for cross-sectional study.

For cut-off point for long working hours, the effects were statistically significant for ‘>50 h/week or >10 h/day’ and ‘≤50 h/week or ≤10 h/day’ (*p* < 0.01), with odds ratios of 1.420 and 1.097, respectively. The workers working more than 50 h per week or more than 10 per day had a higher risk of experiencing occupational health problems than those working 50 or less hours per week or 10 hours or less per day.

For country of origin, the effects were statistically significant for the subgroups of Asian countries and Western countries (*p* < 0.001), with odds ratios of 1.321 and 1.180, respectively. The four subgroups in Asian countries were: ‘China’, ‘China and Japan’, ‘Japan’ and ‘Korea’. The participants in the study conducted by Tayama and Munakata [77] involved both Chinese and Japanese without specifying the proportions. Therefore, this study was regarded as an individual subgroup in the analysis. The effects were statistically significant in the subgroups ‘China’, ‘Japan’ and ‘Korea’ (*p* < 0.001), with odds ratios for the subgroups of 1.745, 1.333 and 1.237, respectively. China showed the strongest effect for long working hours on occupational health. The eight subgroups in the Western countries subgroup were: ‘Australia and New Zealand’, ‘Denmark’, ‘Finland’, ‘Italy’, ‘Spain’, ‘Sweden’, the ‘United Kingdom’ and the ‘United States’. The effects were statistically significant for the subgroups ‘Australia and New Zealand’, ‘Spain’, ‘the United Kingdom’ and ‘the United States’ (*p* < 0.05), with odds ratios of 1.230, 1.248, 1.083 and 1.274, respectively. The United States showed the strongest effect for long working hours on occupational health.

For the health measure category, among the five physiological illnesses in the condition of physical health, the effects were statistically significant for the subgroups ‘cardiovascular heart diseases’ and ‘metabolic syndrome’ (*p* < 0.01), with odds ratios of 1.539 and 1.110, respectively. Long working hours were more strongly associated with cardiovascular heart diseases than with metabolic syndrome. In the related health condition, the effects were statistically significant in the subgroups of fatigue, injury, poor sleep quality, short sleep duration and sleep disturbance (*p* < 0.05), with odds ratios of 1.439, 1.276, 1.276, 1.909 and 1.395, respectively. Out of the five health problems, short sleep duration was the most severe health problem associated with working long hours.

The effects of working class on the relationship between long working hours and each condition (PH, MH, HB, RH and NH) were examined. The meta-regression *p*-values for physiological health, mental health, health behaviours, related health and nonspecified health were 0.485, 0.595, 0.216, 0.001 and 0.161, respectively. The effect of working class on the condition of related health was statistically significant. The effect was statistically significant in the subgroup of blue collar occupations, with an odds ratio of 1.366. Table 4 shows the moderating effect of working class on the association between long working hours and the five conditions (physiological health, mental health, health behaviours, related health and nonspecified health).

## 4. Discussion

This meta-analysis synthesising 243 records from 46 papers with 814,084 participants from 13 countries demonstrated that long working hours had a positive relationship with occupational health problems. The aggregated odds ratio for the effect of long working hours on occupational health was 1.245 (95% CI: 1.195–1.298). Amongst the five occupational health conditions, the condition ‘related health’ showed the strongest association with long working hours; the health measures in this category were short sleep duration, sleep disturbance, sleep problem, exhaustion and injuries.

### 4.1. The Effects of Long Working Hours on Sleep, Fatigue and Injuries

Based on the odds ratios of the five health measures for the condition of related health, short sleep duration had the highest odds ratio and is the problem to be most concerned about with regard to long working hours. Studies referred to in this meta-analysis were concerned with investigating the relationship between long working hours and short sleep duration [8,47,48,78]. The length of hours defined for short sleep was less than 6 h per day by Artazcoz et al. [8] and Ohtsu et al. [78], and less than 7 h per day by Virtanen et al. [48]. These four studies demonstrated that the longer the working hours, the higher risk of suffering from insufficient sleep for both male and female workers. Virtanen et al. [48] found that the odds ratios for both males and females working more than 55 h per week were higher than those working 41 to 55 h per week. Nakashima et al. [47] found that the values for odds ratios between long working hours and short sleep duration were proportional to the relationship with the working hours. Artazcoz et al. [8] showed that the odds ratio between working 51 to 60 h per week and short sleep duration was higher than the odds ratio for working 41 to 50 h per week. Interestingly, this study also showed that female workers had a higher chance of experiencing short sleep duration than male workers when both worked 51 to 60 h per week. The problems suffered by female workers were more severe than for male workers when working long hours. Conversely, the result from Ohtsu [78] conflicted with that from Artazcoz [8] in that male workers tended to experience the problem of short sleep duration more than female workers while working from 9 h to less than 11 h per day. Several studies have reported that the length of sleep for females was longer than for males [79,80,81]. However, some researchers suggested that female workers do more unpaid work than males, for example, housework, which reduces the duration of sleep for female workers [82,83]. The effect of long working hours on short sleep duration based on the gender difference needs to be further investigated. In addition, the studies just investigated the relationship between working hours and sleep hours, which might potentially neglect other causes of short sleep, such as spending much time on leisure and social activities [84]. Research suggests that short sleep duration seriously threatens the health of workers. Many researchers have examined the adverse effects of short sleep duration. Short sleep duration increases the risk of suffering from cardiovascular heart diseases [85], coronary heart diseases [86,87], obesity [88,89,90], hypertension [91] and type 2 diabetes mellitus [92,93]. The consciousness on the importance of sleep of the workers should be enhanced. Fatigue was the items in the condition of related health constituting the second highest value of odds ratio. One of the reasons resulting in fatigue or exhaustion was undoubtedly insufficient sleep [85]. Exhaustion adversely influences the performance of workers and the productivity of organisations [94,95,96,97]. Sleep disturbance was also a health problem yielding a significant effect in the conditions of related health and involves difficulty in falling asleep, insomnia and disorder during different stages of sleep [98]. Sleep disturbance affects the mental health, circadian rhythms and cognitive function of workers [99,100]. In the long-term, sleep disturbance can lead to cardiovascular diseases, obesity and diabetes [101]. The impacts resulting from the problem of sleep are intimately linked with various physiological and mental health problems defined as occupational health problems by the World Health Organization. To ensure the good duration of sleep for workers, guidelines should be provided to publicise the most appropriate sleeping hours as well as to raise public consciousness on the importance of the duration and quality of sleep.

### 4.2. Effects of Moderators

This meta-analysis evaluated the effects of various moderators to identify the associations between such moderators and long working hours, and the consequences for occupational health. Health measure is a significant moderator on the effect sizes of the conditions of physiological health and related health. In the condition of physiological health, a considerable increase was found in the effect size of cardiovascular heart diseases and a slight increase was found in the effect size of metabolic syndrome. This finding means that workers working long hours have a higher risk of suffering from cardiovascular heart diseases and metabolic syndrome compared to those not working long hours. Some studies have demonstrated that the risk of cardiovascular heart diseases was caused by increase in weekly work hours [102,103]. Yu [104] reported that long working hours, especially 60 or more per week, increased the risk of metabolic syndrome. In the condition of related health, a significant increase in the effect sizes of fatigue, injury, poor sleep quality, short sleep duration and sleep disturbance was found. This indicates that workers are more likely to experience health problems when working long hours. This result is consistent with the study of Afonso et al. [30], which demonstrated that longer work hours resulted in poorer sleep quality and more severe sleep disturbance. Similarly, Son et al. [105] reported that the main factor affecting sleepiness was long working hours. Akerstedt et al. [58] noted that long working hours resulted in sleep disturbance and fatigue. Lombardi et al. [106] also pointed out that increase in working hours led to a decrease in daily sleep hours and an increased risk of work-related injury.

The statistically significant effects of study design showed that the method of case-control study was a stronger estimator than cross-sectional study for the impact of working long hours on occupational health. This result may be due to the characteristics of the study methods in terms of the selection of the participants. For a case-control study, a number of participants with certain diseases or situations should be selected as the case group, and a number of participants without the diseases or situations should be selected as the control group. With case-control study design, the associated risk factors and diseases may be promptly identified, and establishment of causation is more powerful than in a cross-sectional study [107]. The problem of cross-sectional studies is the nonrepresentative sampling due to the existence of bias between participants and nonparticipants [107]. Thus, case-control study design yields a stronger association between long working hours and occupational health than cross-sectional study design. The nonsignificant effect of a prospective cohort study may be due to a relatively low bias method of selecting participants.

Country of origin has a significant influence on the overall effect size. It was found that the effects of long working hours on the occupational health of Asian workers were more severe than for Western workers. A few studies have reported that workers in Asian Countries have longer working hours than workers in Western countries [108,109]. Overwork seems to be common in China, Japan, Korea, Singapore, Hong Kong and Taiwan, and it has been reported that many workers in these countries and cities suffer from cardiovascular heart diseases and cerebrovascular diseases due to overwork [110,111,112]. The governments of Japan, Korea and Taiwan have formulated and established a series of regulations to prevent death from overwork, for instance, the implementation of standard working hours [113]. Working hours in these countries became shorter in the wake of the establishment of such standard working hours. However, the definition of standard working hours is still a controversial issue, and many workers have requested that standard working hours should be shorter [114]. Standard working hours have still not been established in many countries [115]. The issue of working hours is unavoidable in the long-term struggle among governments, employers and employees. The establishment of standard working hours should be a first step towards resolving the problem of long working hours.

The cut-off point for long working hours has a significant effect on the overall effect size. Workers working more than 50 h per week or more than 10 h per day had a higher chance of experiencing occupational health problems than those working 50 or less hours per week or 10 or less hours per day. This finding supports the previous study of Virtanen et al. [19] that reported that the risk of coronary heart disease for those working more than 50 h per week was higher than those working 50 h or less per week. However, the odds ratio of working more than 50 h per week or more than 10 h per day in this study was 1.420, which was lower than the odds ratio of 2.37 reported by Virtanen et al. [19]. Further investigation is needed on this odds ratio discrepancy for the effects of long working hours on occupational health. Virtanen et al. [19] only investigated the association between long working hours and coronary heart disease. The meta-analysis reported here covered different types of occupational health illnesses, making comparisons difficult.

There were no significant effects for gender or diagnosis method. This result for diagnosis method agrees with the finding of Kivimäki et al. [22] that diagnosis method had no significant effect on the association of long working hours with health. Here, there was also no significant effect for working class.

For the effect of working class on the association between long working hours and the five conditions (PH, MH, HB, RH and NH), working class only constitutes a significant influence on the effect size of related health (sleep problem, chronic exhaustion, and occupational injury) in which the blue collar occupations had a higher risk of suffering from health problems than white and pink collar occupations. This finding might be due to the low control over own working time [116]. This is an important issue for future research.

### 4.3. Comparison of Previous Meta-Analysis

In a previous meta-analysis conducted by Sparks et al. [5], they found a small positive correlation between long hours of work and health, and the value of the correlation of psychological health was higher than for physiological health. In the study reported here, it was found that long working hours increased the chance of suffering from occupational health problems by 24.3%, and the chance of suffering from the mental health problems was higher than that of suffering physiological health problems. These results were in agreement with the study by Sparks et al. [5]. However, the difference was that this study covered broader health measures than the study by Sparks et al. [5]. This broader coverage may reflect the issue that more occupational health problems were caused by long working hours and reported on in the review period (1998–2018) used here. In addition, it was found in the study reported here that the effect of long working hours on cardiovascular heart diseases had a higher odds ratio of 1.56 (95% CI: 1.344–1.824) than in the study by Kang et al. [21] in 2012 (OR: 1.37; 95% CI: 1.11–1.70). A major difference between the work reported in this analysis and the analysis by Kang et al. [21] was that in this analysis, more studies on the effect of long working hours on cardiovascular heart diseases were involved than were in Kang et al. [21]. This meta-analysis was conducted with the addition of the more recent studies of Fukuoka et al. [9], Virtanen et al. [117], Cheng et al. [13], Jeong et al. [118] and Ma et al. [119]. The increase of odds ratio from this meta-analysis to that of Kang et al. [21] suggests that the risk of suffering from cardiovascular heart diseases for overtime workers has increased in recent years, though more work is required to clarify the issue. The study of Virtanen et al. [117] could not be compared with the study reported here, as coronary heart disease was considered as just one of the diseases in the category of cardiovascular heart diseases in this study. A meta-analysis conducted by Kivimäki et al. [75] examined the relationship between long working hours and the risk of type 2 diabetes by socioeconomic status stratification. Kivimäki et al. [75], reported only that the effect size of the low socioeconomic status group was robust with a risk ratio of 1.29 (95% CI: 1.06–1.17) and null for the high socioeconomic status group. In the study reported here, it was found that the odds ratio of the effect of long working hours on the risk of type 2 diabetes was 0.855 (95% CI: 0.497–1.472). The discrepancy in the results between these two studies might be because 82.6% (19 of 23) of the papers used in the meta-analysis by Kivimäki et al. [75] were unpublished. Further, socioeconomic status includes various demographic variables such as income, educational level and occupation. These variables for socioeconomic status make it difficult to compare results from different studies [120]. For instance, an individual with a high educational level should belong to the high socioeconomic status group. However, a high educational level might not imply a high-income level (high socioeconomic status group). To avoid this contradiction, this study adopted working class instead of socioeconomic status stratification. However, comparing with the meta-analysis conducted by Kivimäki et al. [22], in the study here, coronary heart disease and stroke were categorised in the same group, cardiovascular heart diseases, and thus, no comparisons could be made. Further, 78.26% (18 of 23) of the selected papers in the study of Kivimäki et al. [22] were unpublished, whereas the meta-analysis reported here only included published papers. The reason was that unpublished data involved the privacy concerns of employers and employees and, therefore, the companies and agencies involved had not given permission for the data to be made public.

### 4.4. Theoretical Implications

There is increasing attention on the effects of long working hours on various types of occupational health problems, and a relatively large amount of related publications over the past decade was seen. Compared with the types of health problems caused by long working hours in the meta-analysis of Sparks and Cooper in 1997 [5], a relatively larger amount of different types of health problems caused by long working hours was identified in this meta-analysis. This reflected that long working hours seem to become a perilous act threatening the health of workers. Based on the results of this meta-analysis, the strongest association between long working hours and short sleep duration was found. Interconnected relationships between various types of occupational health problems were shown. Short sleep duration was shown to adversely affect the physical and mental health of workers working for long hours. In addition, gender difference on the sleep duration was also a significant finding in this study. In many countries, male and female workers may have different roles in their daily lives. This can affect the time allocation for their work and other life aspects. There is still a dispute on which gender tends to have a relatively short sleep duration in view of different specific roles between males and females. Further, working class, a commonly neglected factor in past studies, was found to be a significant factor for the difference of length of working hours in this study. It was found that blue collar workers had a higher risk of experiencing the occupational health problems than white and pink collar workers.

### 4.5. Practical Implications

This study provides evidence for governments, employers and employees about the negative effects of long working hours on the health of workers. Governments should clearly recognise the importance of maintaining the health of workers because the productivity of the workforce is what sustains the development and enhancement of society and the economy. Governments should work to establish standard working hours as a useful step towards safeguarding the health and well-being of workers. If no maximum working hours or standard working hours have been established, the health of workers is threatened by the negative health effects of long working hours. Governments should regularly review working hours and monitor the compliance of companies and employers. Furthermore, companies and employers must recognise that their workers regularly work long hours, recognise the effects on occupational health and endeavour to improve the situation. In addition, employees must be made aware of whether they are working long hours and recognise the potential effects on their physical and mental health wellbeing. Short sleep duration and fatigue are strongly associated with long working hours, and if employees regularly get too little sleep (6 h or less per day), or have chronic fatigue syndrome, they should regulate their daily routine to prevent physical or mental deterioration.

### 4.6. Limitations

This meta-analysis had several limitations. The studies included were all in English. Some studies published in other languages satisfied the selection requirement. Future analyses will include studies in other languages to conduct a more generalisable result. Further, the odds ratios in each study were adjusted by different control variables, for instance, age, gender, education, health behaviours and marital status, which might affect the final effect sizes. Therefore, the control variables need to be considered for the extraction of odds ratios in each study. The measurement of health problems was through self-reporting, from clinical examination health reports and annual body checks. For those studies containing self-reported health problems, the result may not be as accurate as for those containing health examinations by clinics or hospitals. Some studies combined the result from male and female workers without stating the proportions. However, different proportions of males and females involved in the data might influence the result. In future studies on the effect of long working hours on occupational health, the genders should be better distinguished and reported. Furthermore, in order to provide a unified result and prevent the deviated effects caused by different work schedules, studies investigating the workers with shift work or night work schedule were excluded, and only studies investigating the daytime workers were included in this meta-analysis. Different work schedules may cause different types and extents of detrimental effects on the occupational health of workers. Focusing on the investigation on one type of work schedule can minimise certain work-schedule biased health effects. Thus, the effects of long working hours on the occupational health of night workers and workers of different work shifts were not considered or evaluated in this meta-analysis. In addition, most studies did not classify the workers into an exhaustive working class. It is difficult to analyse the effect of working class on the relationship between long working hours and occupational health. Therefore, a comprehensive working class is suggested for the future studies investigating this issue. In addition, the global economic crisis has been shown as a significant cause leading to long working hours and overtime among organisations in past studies [121,122]. Thus, the extents and effects of dynamics of economic crisis on the association between long working hours and occupational health can be evaluated in future studies.

## 5. Conclusions

This meta-analysis synthesised 243 records from 46 papers published from 1998 to 2018 to examine the effect of long working hours on the occupational health of workers. Five conditions were classified for occupational health, namely, physiological health, mental health, health behaviours, related health and nonspecified health. The odds ratios and adjustments for publication bias were computed for each condition. The result demonstrated that employees working long hours were vulnerable to suffering from diverse types of occupational health problem. The condition ‘related health’ constituted the highest odds ratio and the health measures included in this condition were short sleep duration, fatigue, sleep disturbance, sleep problem and injury. Workers working long hours had a higher chance of experiencing occupational health problems, and short sleep duration yielded the strongest association with long working hours among the health measures in the related health condition. The findings emphasise the deleterious effects of long working hours on occupational health.

## Figures and Tables

**Table 1:** Characteristics of the 46 papers analysed.

Characteristics	Percentage
Publication years	
1998–2007	26.09
2008–2018	73.91
Origin	
Asian countries	61.59
Western countries	38.41
Gender	
Males	58.73
Females	41.27
Study design	
Case-control study	10.87
Cross-sectional study	54.35
Prospective cohort study	34.78
Diagnosis method	
Self-report	63.04
Health or medical examination	36.96

**Table 2:** Results of meta-analysis between long working hours and occupational health conditions and the adjustment for publication bias.

Occupational Health Condition	Number of Records	Effect Size and 95% Interval			Heterogeneity		Adjustment for Publication Bias			
		Overall OR	Lower Limit	Upper Limit	*p*-Value	I-Squared	Data Points Imputed	Overall OR	Lower Limit	Upper Limit
PH	85	1.177	1.102	1.257	0.000	67.131	6	1.118	1.041	1.200
MH	55	1.366	1.238	1.507	0.000	55.733	12	1.197	1.072	1.336
HB	35	1.100	1.004	1.204	0.000	59.660	0	1.100	1.004	1.204
RH	54	1.465	1.332	1.611	0.000	68.678	7	1.323	1.188	1.473
NH	14	1.065	0.942	1.204	0.001	63.539	0	1.065	0.942	1.204
Overall	243	1.245	1.195	1.298	0.000	67.574				

PH = physiological health, MH = mental health, HB = health behaviours, RH = related health, NH = nonspecified health, OR = odds ratio.

**Table 3:** The association of long working hours with occupational health in relation to gender, diagnosis, study design, cut-off point for long working hours, working class, country of origin and health measure for the conditions of physiological health, mental health, health behaviours, related health and nonspecified health (effect sizes adjusted, when appropriate, for age, gender, educational level and occupation).

Moderator	Effect Size and 95% Interval			Test of Null		Test to Model		
	Odds Ratio	95% Lower	95% Upper	*Z*-Value	2-Sided *p*-Value	*Q*-Value	df (*Q*)	Meta-Regression *p*-Value
Gender						5.797	2.000	0.055
Males	1.280	1.176	1.394	5.711	0.000			
Females	1.135	1.053	1.222	3.332	0.001			
Diagnosis method						1.579	1.000	0.209
Self-report	1.263	1.205	1.324	9.735	0.000			
Health or medical examination	1.188	1.094	1.291	4.086	0.000			
Study design						56.377	2.000	0.000 **
Case-control study **	1.811	1.466	2.239	5.499	0.000			
Cross-sectional study **	1.338	1.267	1.414	10.465	0.000			
Prospective cohort study	1.049	0.997	1.104	1.826	0.068			
Cut-off point for long working hours						57.331	2.000	0.000 **
>50 h/week or >10 h/day **	1.420	1.337	1.508	11.446	0.000			
≤50 h/week or ≤10 h/day **	1.097	1.035	1.162	3.130	0.002			
Working class						1.318	2.000	0.517
White collar occupations	1.095	1.043	1.149	3.668	0.000			
Pink collar occupations	1.168	1.002	1.360	1.992	0.046			
Blue collar occupations	1.275	0.907	1.792	1.400	0.161			
Country of origin						35.043	12.000	0.000 **
Asian Countries **	1.321	1.231	1.418	7.741	0.000			
China **	1.745	1.428	2.132	5.441	0.000			
China and Japan	1.569	0.817	3.013	1.352	0.176			
Japan **	1.333	1.191	1.492	5.010	0.000			
Korea **	1.237	1.124	1.361	4.351	0.000			
Western countries **	1.180	1.126	1.237	6.854	0.000			
Australia and New Zealand *	1.230	1.050	1.442	2.801	0.010			
Denmark	1.091	0.840	1.418	0.656	0.512			
Finland	1.063	0.966	1.170	1.250	0.211			
Italy	1.341	0.993	1.811	1.915	0.055			
Spain *	1.248	1.131	1.377	4.404	0.000			
Sweden	1.198	0.937	1.532	1.438	0.150			
The UK *	1.083	1.008	1.163	2.187	0.029			
The US **	1.274	1.108	1.465	3.393	0.001			
Health measure								
Physiological health						35.773	4.000	0.000 **
All-cause mortality	0.975	0.924	1.029	−0.920	0.358			
Cardiovascular heart diseases **	1.539	1.324	1.789	5.607	0.000			
Metabolic syndrome **	1.100	1.025	1.182	2.630	0.009			
Poor physical health	1.408	0.893	2.221	1.471	0.141			
Type 2 diabetes	0.855	0.497	1.472	−0.565	0.572			
Mental health						5.074	5.000	0.407
Anxiety	1.308	1.041	1.644	2.301	0.021			
Depressive symptoms	1.489	1.220	1.817	3.915	0.000			
Poor mental health	1.239	1.018	1.510	2.134	0.033			
Psychiatric morbidity	1.398	1.184	1.651	3.952	0.000			
Psychological distress	1.110	0.878	1.403	0.870	0.384			
Psychological stress	1.512	1.123	2.034	2.727	0.006			
Health behaviours						2.255	3.000	0.521
Heavy drinking	1.083	0.943	1.244	1.134	0.257			
Physical inactivity	1.234	1.002	1.520	1.978	0.048			
Smoking	1.055	0.890	1.251	0.620	0.535			
Unhealthy food habits	0.990	0.796	1.230	−0.094	0.925			
Related health						9.604	4.000	0.048 *
Fatigue **	1.439	1.149	1.803	3.169	0.002			
Injury **	1.276	1.091	1.492	3.047	0.002			
Poor sleep quality **	1.276	1.128	1.444	3.880	0.000			
Short sleep duration **	1.909	1.502	2.427	5.281	0.000			
Sleep disturbance *	1.395	1.052	1.850	2.312	0.021			
Nonspecified health						-	-	-
Poor health status	1.065	0.942	1.204	1.000	0.317			

** *p*-value < 0.01. * *p*-value < 0.05.

**Table 4:** Moderating effect of working class on the association of long working hours with physiological health, mental health, health behaviours, related health and nonspecified health (effect sizes adjusted, when appropriate, for age, gender, educational level and occupation).

Working Class	Odds Ratio	95% Lower	95% Upper	*Z*-Value	2-Sided *p*-Value	*Q*-Value	df (*Q*)	Meta-Regression *p*-Value
Physiological health						1.449	2.000	0.485
White collar occupations	1.145	1.007	1.303	2.065	0.039			
Pink collar occupations	0.986	0.792	1.226	−0.130	0.896			
Blue collar occupations	1.192	0.747	1.902	0.737	0.461			
Mental health						1.037	2.000	0.595
White collar occupations	1.310	1.166	1.473	4.546	0.000			
Pink collar occupations	1.760	0.961	3.223	1.831	0.067			
Blue collar occupations	1.250	0.962	1.624	1.672	0.095			
Health behaviours						3.069	2.000	0.216
White collar occupations	0.988	0.915	1.066	−0.316	0.752			
Pink collar occupations	1.102	0.745	1.629	0.487	0.626			
Blue collar occupations	1.250	0.962	1.624	1.672	0.095			
Related health						13.143	2.000	0.001 *
White collar occupations	0.887	0.713	1.104	−1.075	0.282			
Pink collar occupations	0.989	0.940	1.040	−0.438	0.662			
Blue collar occupations *	1.366	1.144	1.631	3.445	0.001			
Nonspecified health						3.649	2.000	0.161
White collar occupations	0.970	0.853	1.103	−0.463	0.643			
Pink collar occupations	0.881	0.666	1.165	−0.890	0.374			
Blue collar occupations	1.115	0.987	1.260	1.745	0.081			

* *p*-value < 0.01.

## Supplementary Materials

The following are available online at https://www.mdpi.com/1660-4601/16/12/2102/s1, Table S1: The information and odds ratios of the 46 papers (sorted in year of publication).
