# Supplementary material for: The Effect of Long Working Hours and Overtime on Occupational Health: A Meta-Analysis of Evidence from 1998 to 2018
Source: Int J Environ Res Public Health. 2019 Jun 13;16(12):2102. doi: 10.3390/ijerph16122102 (PMC6617405; doi:10.3390/ijerph16122102)
Supplement: Supplementary file 1 [file ijerph-16-02102-s001.pdf]

Table S1. The information and odds ratios of the 46 papers (sorted in year of publication).

First author (published year)	Country of origin	Number of participants	Gender	Study design	Diagnosis method	Working class	Health measure	Reference working hours	Working hours	Occupational health condition	OR	95% CI
Sokejima (1998)[a] [1]	Japan	526	Male	Case-control study	HME	Yes	Acute myocardial infarction	≤7-9h/d	9-11h/d	PH	0.960	0.578-1.594
									>11h/d	PH	2.940	0.139-6.234
Nakanishi (2001)[a] [2]	Japan	1266	Male	Prospective cohort study (5 years)	HME	Yes	Type 2 diabetes	<8h/d	8-8.9h/d	PH	0.947	0.496-1.808
									9-9.9h/d	PH	0.447	0.177-1.132
									10-10.9h/d	PH	0.469	0.173-1.272
									≥11h/d	PH	0.375	0.126-1.116
Nakanishi (2001)[a] [3]	Japan	941	Male	Prospective cohort study (5 years)	HME	Yes	Hypertension	<8h/d	8-8.9h/d	PH	0.769	0.435-1.361
									9-9.9h/d	PH	1.001	0.550-1.823
									10-10.9h/d	PH	0.534	0.254-1.123
									≥11h/d	PH	0.223	0.077-0.647
Liu (2002)[a] [4]	Japan	705	Male	Case-control study	HME	Yes	Acute myocardial infarction	≤40 h/w	41-60h/w	PH	-	-
									>61h/w	PH	1.800	0.991-3.270
Akerstedt (2002)[a] [5]	Sweden	58115 (M:30524; F:27591)	Male and female	Prospective cohort study (20 years)	SR	Yes	Disturbed sleep	≤50 h/w	>50 h/w	RH	1.050	0.911-1.211
							Fatigue		>50 h/w	RH	1.350	1.231-1.481
Tarumi (2003) [6]	Japan	867 (M:778; F:89)	Male and female	Prospective cohort study (3.6 years)	SR	Yes	Mental disorders	≤44h/w	45-49.9h/w	MH	1.333	0.442-4.013
									≥50h/w	MH	1.321	0.439-3.980
							Circulatory disorders		45-49.9h/w	PH	1.553	0.744-3.240
									≥50h/w	PH	1.220	0.560-2.655
Fukuoka (2005)[a] [7]	Japan	94 (M:92; F:2)	Male and female	Cross-sectional study	HME	Yes	Acute myocardial infarction	<55h/w	≥55h/w	PH	0.870	0.236-3.206
Uchiyama (2005)[a] [8]	Japan	1615 (M:908; F:707)	Male	Prospective cohort study (5.6 years)	HME	Yes	Cardiovascular heart disease	≤9h/d	≥10h/d	PH	1.204	0.561-2.582
			Female						≥10h/d	PH	0.388	0.022-6.727
Grosch (2006)[a] [9]	The US				SR	Yes	Job stress	≤40h/w	41-48h/w	MH	1.160	0.840-1.601
									49-69h/w	MH	1.790	1.351-2.372

		1744 (M:849; F:895)	Male and female	Cross-sectional study					≥70h/w	MH	2.490	1.561-3.972
							Hurt		41-48h/w	RH	0.860	0.515-1.436
									49-69h/w	RH	1.240	0.810-1.899
									≥70h/w	RH	1.870	0.993-3.521
							General Health		41-48h/w	NH	0.900	0.570-1.421
									49-69h/w	NH	0.540	0.338-0.864
									≥70h/w	NH	1.940	1.071-3.514
							Poor mental health		41-48h/w	MH	1.050	0.671-1.643
									49-69h/w	MH	1.020	0.676-1.540
									≥70h/w	MH	1.590	0.851-2.972
							Poor physical health		41-48h/w	PH	0.820	0.429-1.567
									49-69h/w	PH	0.740	0.398-1.374
									≥70h/w	PH	5.020	2.488-10.130
Sekine (2006)[a] [10]	Japan	3556 (M:2397; F:1159)	Male	Cross-sectional study	SR	Yes	Poor sleep quality	7-9h/d	9-11h/d	RH	1.110	0.839-1.468
									<11h/d	RH	1.490	1.031-2.153
			Female						9-11h/d	RH	1.320	0.948-1.837
									<11h/d	RH	2.020	1.236-3.300
Yang (2006)[a] [11]	The US	24205 (M:13724; F:10481)	Male and female	Cross-sectional study	SR	Yes	Hypertension	11-39h/w	41-50h/w	PH	1.170	1.035-1.323
									≥51h/w	PH	1.290	1.097-1.516
Nagashima (2007)[a] [12]	Japan	715	Male	Cross-sectional study	SR	No	Fatigue	<50h/w	50-<50h/w	RH	0.900	0.570-1.421
									<55h/w-<60h/w	RH	0.810	0.450-1.459
									60-<65h/w	RH	1.070	0.586-1.955
									65-<70h/w	RH	1.890	0.983-3.632
									≥70h/w	RH	2.320	1.208-4.454
							Physical disorder		50-<50h/w	PH	1.410	0.821-2.422
									<55h/w-<60h/w	PH	1.090	0.541-2.195
									60-<65h/w	PH	0.770	0.321-1.846
									65-<70h/w	PH	1.850	0.832-4.112
									≥70h/w	PH	2.430	1.103-5.354

							Anxiety		50-<50h/w	MH	1.110	0.709-1.738
									<55h/w-<60h/w	MH	0.910	0.509-1.627
									60-<65h/w	MH	1.210	0.660-2.219
									65-<70h/w	MH	2.280	1.198-4.341
									≥70h/w	MH	2.510	1.288-4.892
							Chronic tiredness		50-<50h/w	RH	1.290	0.760-2.190
									<55h/w-<60h/w	RH	1.140	0.584-2.225
									60-<65h/w	RH	1.500	0.750-3.000
									65-<70h/w	RH	3.570	1.782-7.150
									≥70h/w	RH	2.850	1.361-5.966
Lallukka (2008)[a] [13]	London, the UK	3397	Male	Prospective-sectional study (2 years)	SR	Yes	Unhealthy food habits	≤40h/w	>40h/w	HB	0.780	0.587-1.036
							Physical inactivity			HB	0.970	0.754-1.247
							Heavy drink			HB	1.150	0.862-1.534
							Smoking			HB	1.100	0.859-1.408
							Obesity			PH	1.110	0.810-1.521
			Female				Unhealthy food habits			HB	1.000	0.610-1.640
							Physical inactivity			HB	0.810	0.473-1.388
							Heavy drink			HB	0.950	0.606-1.490
							Smoking			HB	1.290	0.813-2.047
							Obesity			PH	1.710	1.115-2.622
	Helsinki, Finland	6067	Male				Unhealthy food habits			HB	1.300	0.892-1.894
							Physical inactivity			HB	1.140	0.748-1.736
							Heavy drink			HB	1.640	0.940-2.861
							Smoking			HB	0.730	0.489-1.089
							Obesity			PH	1.280	0.822-1.994

			Female				Unhealthy food habits			HB	1.030	0.774-1.370
							Physical inactivity			HB	1.050	0.836-1.319
							Heavy drink			HB	0.980	0.706-1.361
							Smoking			HB	1.030	0.834-1.271
							Obesity			PH	1.080	0.854-1.366
	Japan	2213	Male				Physical inactivity			HB	0.980	0.681-1.411
							Heavy drink			HB	0.830	0.584-1.179
							Smoking			HB	0.670	0.494-0.908
							Obesity			PH	0.860	0.611-1.211
			Female				Physical inactivity			HB	0.950	0.490-1.841
							Heavy drink			HB	0.770	0.227-2.609
							Smoking			HB	1.470	0.341-6.343
							Obesity			PH	0.640	0.350-1.170
Artazcoz (2009)[a] [14]	Spain	7085 (M: 3950; F:3135)	Male	Cross-sectional study	SR	Yes	Poor self-perceived health status	30-40h/w	41-50h/w	NH	0.960	0.747-1.234
									51-60h/w	NH	1.430	0.977-2.093
							Poor mental health status		41-50h/w	MH	1.240	0.905-1.700
									51-60h/w	MH	2.060	1.310-3.240
							Hypertension		41-50h/w	PH	1.190	0.947-1.495
									51-60h/w	PH	1.600	1.119-2.288
							Smoking		41-50h/w	HB	1.110	0.953-1.293
									51-60h/w	HB	1.330	1.029-1.719
							Physical inactivity		41-50h/w	HB	1.730	1.314-2.277
									51-60h/w	HB	2.430	1.640-3.600
							Insufficient sleep		41-50h/w	RH	1.300	1.111-1.521
									51-60h/w	RH	1.420	1.090-1.850
			Female				Poor self-perceived health status		41-50h/w	NH	1.020	0.749-1.389
									51-60h/w	NH	1.380	0.743-2.562
							Poor mental health status		41-50h/w	MH	0.990	0.728-1.346
									51-60h/w	MH	1.430	0.771-2.653
							Hypertension		41-50h/w	PH	0.660	0.426-1.021
									51-60h/w	PH	1.060	0.458-2.451

							Smoking		41-50h/w	HB	0.840	0.663-1.065
									51-60h/w	HB	2.270	1.391-3.704
							Physical inactivity		41-50h/w	HB	1.380	0.961-1.982
									51-60h/w	HB	1.530	0.690-3.391
							Insufficient sleep		41-50h/w	RH	1.000	0.781-1.281
									51-60h/w	RH	2.210	1.343-3.637
Pimenta (2009)[a] [15]	Spain	8779 (M:3528; F:5251)	Male	Prospective cohort study (4.2 years)	SR	Yes	Hypertension	≤39h/w	40-49h/w	PH	1.030	0.746-1.422
									50-60h/w	PH	1.030	0.754-1.407
									≥60h/w	PH	0.980	0.633-1.517
			Female						40-50h/w	PH	1.220	0.760-1.959
									≥50h/w	PH	1.080	0.590-1.978
Virtanen (2009) [16]	London, the UK	2470 (M:1872; F:598)	Male and female	Prospective cohort study (3 years)	SR	Yes	Insufficient sleep (<7h/d)	35-40h/w	41-55h/w	RH	1.550	1.031-2.331
									>55h/w	RH	2.800	1.200-6.532
Driesen (2010)[a] [17]	Italy	7217	Male	Cross-sectional study	SR	No	Depressive symptoms	<45h/w	45-50h/w	MH	1.250	0.845-1.850
									51-60h/w	MH	1.420	0.779-2.588
									>60/w	MH	2.360	0.752-7.408
			Female						>45h/w	MH	1.180	0.440-3.162
Holtermann (2010)[a] [18]	Copenhagen, Denmark	4964	Male	Prospective cohort study (30 years)	HME	No	Ischaemic heart disease	≤40h/w	45-49.5h/w	PH	1.610	1.197-2.164
									≥50h/w	PH	1.257	0.886-1.783
							All-cause mortality	≤40h/w	45-49.5h/w	PH	1.055	0.890-1.250
									≥50h/w	PH	0.789	0.644-0.966
Nash (2010)[a] [19]	Australia	2953 (M:2085; F:868)	Male and female	Cross-sectional study	SR	Yes	Psychiatric morbidity	<40h/w	40-49h/w	MH	1.230	0.932-1.623
									50-59h/w	MH	1.410	1.051-1.892
									≥60h/w	MH	1.650	1.202-2.264
							Hazardous alcohol use	<40h/w	40-49h/w	HB	1.130	0.809-1.578
									50-59h/w	HB	1.150	0.818-1.616
									≥60h/w	HB	0.670	0.452-0.994
Netterstrøm (2010)[a] [20]	Copenhagen, Denmark	1146 (M:551; F:595)	Male	Prospective cohort study (14 years)	HME	Yes	Ischaemic heart disease	<50h/w	>50h/w	PH	0.700	0.191-2.572
			Female					<50h/w	>50h/w	PH	0.800	0.091-7.065
Virtanen (2010) [21]	The UK	6014 (M:4262; F:1752)	Male and female	Prospective cohort study (11 years)	SR	Yes	Coronary heart disease	7-8 h/d	9h/d	PH	0.951	0.716-1.262
									10h/d	PH	1.167	0.865-1.576
									11-12h/d	PH	1.462	1.060-2.018

Kivimäki (2011) [22]	London, the UK	7095 (M:4986; F:2109)	Male and female	Prospective cohort study (12.3 years)	HME	No	Coronary heart disease	<11h/d	≥11h/d	PH	1.670	1.097-2.543
Nakashima (2011)[a] [23]	Japan	1510	Male	Cross-sectional study	SR	Yes	Poor sleep quality	<46.5h/w	≥46.5-<50h/w	RH	0.900	0.600-1.350
									≥50-<52.5h/w	RH	1.220	0.826-1.802
									≥52.5-<57.75h/w	RH	1.380	0.937-2.033
									≥57.75h/w	RH	1.380	0.929-2.049
							Short sleep duration		≥46.5-<50h/w	RH	1.430	1.024-1.997
									≥50-<52.5h/w	RH	1.510	1.080-2.111
									≥52.5-<57.75h/w	RH	1.750	1.250-2.450
									≥57.75h/w	RH	3.680	2.587-5.234
Virtanen (2011)[a] [24]	The UK	2960 (M:2248; F: 712)	Male	Prospective cohort study (5 years)	SR	Yes	Depression	≤40h/w	41-55h/w	MH	0.844	0.618-1.152
									>55h/w	MH	1.035	0.624-1.719
							Anxiety		41-55h/w	MH	0.947	0.704-1.275
									>55h/w	MH	1.228	0.769-1.962
			Female				Depression		41-55h/w	MH	1.855	1.117-3.082
									>55h/w	MH	1.874	0.770-4.559
							Anxiety		41-55h/w	MH	1.316	0.819-2.113
									>55h/w	MH	2.093	0.967-4.533
Kobayashi (2012)[a] [25]	Japan	911	Male	Cross-sectional study	HME	Yes	Metabolic syndrome	≤7 to 8 h/d	>8-9h/d	PH	1.660	0.913-3.019
									>9-10h/d	PH	1.480	0.753-2.910
									>10h/d	PH	2.320	1.042-5.168
Schluter (2012)[a] [26]	Australia and New Zealand	4419 (M:3552; F:867)	Male and female	Cross-sectional study	SR	Yes	Alcohol use	<40h/w	40-49h/w	HB	1.260	1.006-1.579
									≥50h/w	HB	1.460	1.020-2.090
Virtanen (2012)[a] [27]	London, the UK	2123 (M:1626; F:497)	Male and female	Prospective Cohort Study (5 years)	SR	Yes	Depressive symptoms	7-8h/d	9h/d	MH	0.620	0.279-1.376
									10h/d	MH	1.180	0.557-2.498
									11-12/d	MH	2.300	1.048-5.049
Wirtz (2012)[a] [28]	The US	96915 (M: 48816; F:48099)	Male	Cross-sectional study	HME	Yes	Injury	31-40h/w	41/50h/w	RH	1.210	0.923-1.586
									>50h/w	RH	1.120	0.799-1.569
			Female						41/50h/w	RH	1.510	1.031-2.212

									>50h/w	RH	1.690	1.059-2.697
Jang (2013)[a] [29]	Korea	8889 (M:5241; F:3648)	Male	Cross-sectional study	HME	Yes	Obesity	40-48h/w	49-60h/w	PH	1.115	0.856-1.452
									>60h/w	PH	1.647	1.262-2.150
									49-60h/w	PH	0.878	0.650-1.186
									>60h/w	PH	1.059	0.699-1.604
			Female						49-60h/w	PH	0.961	0.687-1.345
									>60h/w	PH	0.863	0.624-1.194
									49-60h/w	PH	1.340	0.781-2.299
									>60h/w	PH	0.916	0.363-2.310
Jeong (2013)[a] [30]	Korea	1117 (M:926; F:121)	Male and female	Case-control study	HME	Yes	Cardiovascular heart disease	40-48h/w	48.1-52h/w	PH	1.730	1.031-2.903
									>52h/w	PH	3.460	2.380-5.030
Lee (2013)[a] [31]	Korea	561 (M:417; F:144)	Male and female	Cross-sectional study	SR	No	Low mood	35-47h/w	48-52h/w	MH	0.820	0.530-1.260
									53-60h/w		1.260	0.780-2.050
									≥60h/w		1.030	0.410-2.580
							Depression		48-52h/w	MH	1.36	0.820-2.260
									53-60h/w		2.94	1.540-5.590
									≥60h/w		4.35	2.180-8.67
Ohtsu (2013)[a] [32]	Japan	662 (M:372; F:290)	Male	Cross-sectional study	SR	No	Short sleep (<6h/d)	7-<9h/d	≥9-<11h/d	RH	2.760	1.569-4.856
									≥11h/d	RH	8.620	3.940-18.859
			Female						≥9-<11h/d	RH	2.510	1.169-5.387
O' Reilly (2013)[a] [33]	North Ireland, the UK	414949 (M:270011; F:144938)	Male	Prospective Cohort Study (8.7 years)	HME	Yes	All-cause mortality	35-40h/w	41-48h/w	PH	0.960	0.879-1.049
									49-54h/w	PH	1.020	0.920-1.130
									≥55h/w	PH	0.970	0.880-1.070
			Female						41-48h/w	PH	0.980	0.797-1.205
									49-54h/w	PH	1.170	0.871-1.572
									≥55h/w	PH	0.860	0.631-1.172
Cheng (2014)[a] [34]	Taiwan, China	966	Male	Case-control study	HME	No	Acute myocardial infarction	40-48h/w	>48-60h/w	PH	1.600	1.012-2.530
									>60h/w	PH	2.400	1.470-3.919
									>48-60h/w	PH	1.600	1.209-2.117
									>60h/w	PH	2.300	1.703-3.106
Tayama (2014)[a] [35]	China and Japan	2228 (M:1426; F:802)	Male	Cross-sectional study	HME	Yes	Diabetes	<45 h/w	45-54h/w	PH	1.130	0.736-1.735
									≥55h/w	PH	2.200	1.391-3.479
			Female						45-54h/w	PH	-	-
									≥55h/w	PH	-	-

Bannai (2015)[a] [36]	Japan	515 (M:335; F:180)	Male	Cross-sectional study	SR	Yes	Sleep problem	≤40h/w	>40-≤50h/w	RH	1.140	0.470-2.768
									>50-≤60h/w	RH	1.200	0.547-2.634
									>60h/w	RH	2.050	0.994-4.230
			Female						>40-≤50h/w	RH	0.640	0.169-2.430
									>50-≤60h/w	RH	0.650	0.187-2.262
									>60h/w	RH	1.260	0.365-4.345
Cho (2015)[a] [37]	Korea	1578 (M:1072; F:506)	Male	Prospective cohort study (7 years)	SR	Yes	Poor self-rated health	36-40h/w	41-52h/w	NH	0.890	0.745-1.063
									52-68h/w	NH	0.930	0.754-1.147
									≥69h/w	NH	1.020	0.787-1.322
			Female				Poor self-rated health		41-52h/w	NH	1.000	0.767-1.304
									52-68h/w	NH	1.170	0.853-1.605
									≥69h/w	NH	1.630	1.050-2.530
Hino (2015) [38]	Japan	1198 (M:457; F:741)	Male and female	Cross-sectional study	SR	Yes	Psychological distress	≤55h/w	>45-79h/w	MH	1.260	0.800-1.980
									>80h/w	MH	1.380	0.730-2.600
Kim (2015)[a] [39]	Korea	34783 (M: 20543; F: 14240)	Male	Cross-sectional study	SR	Yes	Sleep disturbance	≥40-<48h/w	≥49-≤60h/w	RH	2.389	1.807-3.158
									>60h/w	RH	3.017	1.956-4.653
									≥49-≤60h/w	RH	1.041	0.766-1.415
									>60h/w	RH	1.418	1.003-2.005
			Female						≥49-≤60h/w	RH	1.525	1.034-2.249
									>60h/w	RH	0.994	0.398-2.484
									≥49-≤60h/w	RH	0.826	0.540-1.263
									>60h/w	RH	1.241	0.721-2.136
Kim (2016)[a] [40]	South Korea	6805 (M:4319; F:2486)	Male and female	Cross-sectional study	SR	No	Depressive symptoms	35-40h/w	41-52h/w	MH	0.770	0.542-1.094
									53-68h/w	MH	1.000	0.743-1.346
									>68h/w	MH	1.570	1.052-2.344
Yoon (2016)[a] [41]	Korea	42234	Female	Cross-sectional study	HME	Yes	Obesity	<40h/w	50-54h/w	PH	1.080	0.859-1.358
									55-59h/w	PH	1.020	0.724-1.437
									≥60h/w	PH	1.280	1.042-1.573

			Female						50-54h/w	PH	0.990	0.869-1.128
									55-59h/w	PH	1.110	0.966-1.275
									≥60h/w	PH	1.080	0.859-1.358
Lee (2017)[a] [42]	South Korea	1030	Male	Cross-sectional study	SR	Yes	Psychological stress	40-44 h/w	45-49h/w	MH	0.940	0.520-1.700
									50-54h/w	MH	0.990	0.533-1.840
									55-59h/w	MH	1.330	0.666-2.655
									≥60 h/w	MH	3.000	1.381-6.516
Ma (2017)[a] [43]	China	595 (M:406; F:189)	Male and female	Case-control study	SR	No	Cardiovascular heart disease	Not given	35-40h/w	PH	0.977	0.544-1.754
									41-48h/w	PH	1.439	0.759-2.729
									49-54h/w	PH	1.383	0.669-2.858
									≥55h/w	PH	2.213	1.125-4.354
Nakata (2017) [44]	Japan	2375 (M:1739; F:636)	Male and female	Cross-sectional study	SR	Yes	Depressive symptoms	6-8h/d	>8-10h/d	MH	1.120	0.893-1.405
									>10h/d	MH	1.490	1.069-2.077
Cho (2018)[a] [45]	Korea	32867 (M:20200; F:12667)	Male and female	Cross-sectional study	SR	Yes	Poor self-rated health	≤52h/w	>52h/w	NH	1.300	1.220-1.400
Ogawa (2018)[a] [46]	Japan	973 (M:812; F:161)	Male and female	Cross-sectional study	SR	Yes	Depressive symptoms	<60h/w	60-<80h/w	MH	1.900	0.911-3.962
									80-100h/w	MH	2.830	1.361-5.884
									≥100h/w	MH	6.960	3.048-15.891

Note. [a]: The study was included in the moderator analysis for gender

OR = odds ratio; PH = physiological health; MH = mental health; HB = health behaviours; RH = related health; NH = non-specified health; M = male; F = female; CI = confidence interval; N/A = not applicable; h = hours; w = week; d = day; SR = self-report; HME = health or medical examination

References

1. Sokejima, S.; Kagamimori, S. Working hours as a risk factor for acute myocardial infarction in Japan: Case-control study. *BMJ* **1998**, *317*, 775-780.
2. Nakanishi, N.; Nishina, K.; Yoshida, H.; Matsuo, Y.; Nagano, K.; Nakamura, K.; Suzuki, K.; Tatara, K. Hours of work and the risk of developing impaired fasting glucose or type 2 diabetes mellitus in Japanese male office workers. *Occup. Environ. Med.* **2001**, *58*, 569–574.
3. Nakanishi, N.; Yoshida, H.; Nagano, K.; Kawashimo, H.; Nakamura, K.; Tatara, K. Long working hours and risk for hypertension in Japanese male white collar workers. *J. Epidemiol. Community Health* **2001**, *55*, 316–322.
4. Liu, Y.; Tanaka, H. Overtime work, insufficient sleep, and risk of non-fatal acute myocardial infarction in Japanese men. *Occup. Environ. Med.* **2002**, *59*, 447–451.

5. Akerstedt, T.; Knutsson, A.; Westerholm, P.; Theorell, T.; Alfredsson, L.; Kecklund, G. Sleep disturbances, work stress and work hours: A cross-sectional study. *J. Psychosom. Res.* **2002**, *53*, 741–748.
6. Tarumi, K.; Hagihara, A.; Morimoto, K. A prospective observation of onsets of health defects associated with working hours. *Ind. Health* **2003**, *41*, 101–108.
7. Fukuoka, Y.; Dracup, K.; Froelicher, E.S.; Ohno, M.; Hirayama, H.; Shiina, H.; Kobayashi, F. Do Japanese workers who experience an acute myocardial infarction believe their prolonged working hours are a cause? *Int. J. Cardiol.* **2005**, *100*, 29–35.
8. Uchiyama, S.; Kurasawa, T., Sekizawa, T., Nakatsuka, H. Job strain and risk of cardiovascular events in treated hypertensive Japanese workers: Hypertension follow-up group study. *J. Occup. Health* **2005,** *47*, 102–111.
9. Grosch, J.W.; Caruso, C.C.; Rosa, R.R.; Sauter, S.L. Long hours of work in the US: Associations with demographic and organizational characteristics, psychosocial working conditions, and health. *Am. J. Ind. Med.* **2006**, *49*, 943–952.
10. Sekine, M., Chandola, T., Martikainen, P., Marmot, M., Kagamimori, S. Work and family characteristics as determinants of socioeconomic and sex inequalities in sleep: The Japanese civil servants study. *Sleep* **2006**, *29*, 206–216.
11. Yang, Y.; Schnall, P.L.; Jauregui, M.; Su, T.C.; Baker, D. Work hours and self-reported hypertension among working people in California. *Hypertension* **2006**, *48*, 744–750.
12. Nagashima, S.; Suwazono, Y.; Okubo, Y. Working hours and mental and physical fatigue in Japanese workers. *Occup. Med.* **2007**, *57*, 449–452.
13. Lallukka, T.; Lahelma, E.; Rahkonen, O.; Roos, E.; Laaksonen, E.; Martikainen, P.; Head, J.; Brunner, E.; Mosdol, A.; Marmot, M.; et al. Associations of job strain and working overtime with adverse health behaviors and obesity: Evidence from the Whitehall II Study, Helsinki Health Study, and the Japanese Civil Servants Study. *Soc. Sci. Med.* **2008**, *66*, 1681–1698.
14. Artazcoz, L.; Cortès, I.; Escribà-Agüir, V.; Cascant, L.; Villegas, R. Understanding the relationship of long working hours with health status and health-related behaviours. *J. Epidemiol. Community Health* **2009**, *63*, 521–527.
15. Pimenta, A.M.; Beunza, J.J.; Bes-Rastrollo, M.; Alonso, A.; López, C.N, Velásquez-Meléndez G, Martínez-González M.A. Work hours and incidence of hypertension among Spanish University graduates: The Seguimiento Universidad de Navarra prospective cohort. *J. Hypertens.* **2009**, *27*, 34–40.
16. Virtanen, M.; Ferrie, J.E.; Gimeno, D.; Vahtera, J.; Elovainio, M.; Singh-Manoux, A. Long working hours and sleep disturbances: The Whitehall II prospective cohort study. *Sleep* **2009**, *32*, 737–745.
17. Driesen, K.; Jansen, N.W.; Kant, I.; Mohren, D.C.; van Amelsvoort, L.G. Depressed mood in the working population: Associations with work schedules and working hours. *Chronobiol. Int.* **2010**, *27*, 1062–1079.
18. Holtermann, A.; Mortensen, O.S.; Burr, H.; Søgaard, K.; Gyntelberg, F.; Suadicani, P. Physical demands at work, physical fitness, and 30-year ischaemic heart disease and all-cause mortality in the Copenhagen male study. *Scand. J. Work. Environment. Health* **2010**, *36*, 357–365.
19. Nash, L.M.; Daly, M.G.; Kelly, P.J.; van Ekert, E.H.; Walter, G.; Walton, M.; Willcock ,S.M.; Tennant C.C. Factors associated with psychiatric morbidity and hazardous alcohol use in Australian doctors. *Med. J. Aust.* **2010**, *193*, 161–166.
20. Netterstrøm, B.; Kristensen, T.S.; Jensen, G.; Schnor, P. Is the demand-control model still a usefull tool to assess work-related psychosocial risk for ischemic heart disease? Results from 14 year follow up in the Copenhagen City heart study. *Int. J. Occup. Med. Environ. Health* **2010**, *23*, 217–524.
21. Virtanen, M.; Ferrie, J.E.; Singh-Manoux, A.; Shipley, M.J.; Vahtera, J., Marmot, M.G.; Kivimäki, M. Overtime work and incident coronary heart disease: The Whitehall II prospective cohort study. *Eur. Heart J.* **2010**, *31*, 1737–1744.

22. Kivimäki, M.; Batty, G.D.; Ferrie, J.E.; Vahtera, J.; Virtanen, M., Marmot, M.G.; Singh-Manoux, A; Shipley, M.J. Using additional information on working hours to predict coronary heart disease: A cohort study. *Ann. Intern. Med.* **2011**, *154*, 457–463.
23. Nakashima, M.; Morikawa, Y.; Sakurai, M.; Nakamura, K.; Miura, K.; Ishizaki, M., Kido, T.; Naruse, Y.; Suwazono, Y.; Nakagawa, H. Association between long working hours and sleep problems in white-collar workers. *J. Sleep Res.* **2011**, *20*, 110–116
24. Virtanen, M.; Ferrie, J.E.; Singh-Manoux, A.; Shipley, M.J.; Stansfeld, S.A.; Marmot, M.G.; Ahola, K.; Vahtera, J.; Kivimäki, M. Long working hours and symptoms of anxiety and depression: A 5-year follow-up of the Whitehall II study. *Psychol. Med.* **2011**, *41*, 2485–2494.
25. Kobayashi, T.; Suzuki, E.; Takao, S.; Doi, H. Long working hours and metabolic syndrome among Japanese men: A cross- sectional study. *BMC Public Health* **2012**, *12*, 395, doi:10.1186/1471-2458-12-395.
26. Schluter, P.J.; Turner, C.; Benefer, C. Long working hours and alcohol risk among Australian and New Zealand nurses and midwives: A cross-sectional study. *Int. J. Nurs. Stud.* **2012**, *49*, 701–709.
27. Virtanen, M.; Stansfeld, S.A.; Fuhrer, R.; Ferrie, J.E.; Kivimäki, M. Overtime work as a predictor of major depressive episode: a 5-year follow-up of the Whitehall II study. *PLoS One*. **2012**, *1*, e30719, doi: 10. 0.1371/journal.pone.0030719.
28. Wirtz, A.; Lombardi, D.A.; Willetts, J.L.; Folkard, S.; Christiani, D.C. Gender differences in the effect of weekly working hours on occupational injury risk in the United States working population. *Scand. J. Work Environ. Health* **2012**, *38*, 349–357.
29. Jang, T.W.; Kim, H.R.; Lee, H.E.; Myong, J.P.; Koo, J.W. Long work hours and obesity in Korean adult workers. *J. Occup. Health* **2013**, *55*, 359–366.
30. Jeong, I.; Rhie, J.; Kim, I.; Ryu, I.; Jung, P.K.; Park, Y.S., Lim, Y.S.; Kim, S.G.; Park, S.G.; Im, H.L.; et al. Working hours and cardiovascular disease in Korean workers: A case-control study. *J. Occup. Health* **2013**, *55*, 385-391.
31. Lee, K.H.; Kim, J.E.; Kim, Y.K.; Kang, D.M.; Yun, M.Y.; Park, S.G.; Song, J.S.; Lee, S.G. Long working hours and emotional well-being in Korean manufacturing industry employees. *Ann. Occup. Environ. Med.* **2013**, 25, 38, doi:10.1186/2052-4374-25-38.
32. Ohtsu, T.; Kaneita, Y.; Aritake, S.; Mishima, K.; Uchiyama, M.; Akashiba, T., Uchimura, N.; Nakaji, S.; Munezawa, T.; Kokaze, A.; et al. A cross-sectional study of the association between working hours and sleep duration among the Japanese working population. *J. Occup. Health* **2013**, *4*, 307–311.
33. O' Reilly, D.; Rosato, M. Worked to death? A census-based longitudinal study of the relationship between the numbers of hours spent working and mortality risk. *Int. J. Epidemiol.* **2013**, *42*, 1820–1830.
34. Cheng, Y.; Du, C.L.; Hwang, J.J.; Chen, I.S.; Chen, M.F.; Su, T.C. Working hours, sleep duration and the risk of Acute Coronary Heart Disease: A case-control study of middle aged men in Taiwan. *Int. J. Cardiol.* **2014**, *171*, 419–422.
35. Tayama, J.; Li, J.; Munakata, M. Working long hours is associated with higher prevalence of diabetes in urban male Chinese workers: The rosai karoshi study. *Stress Health* **2014**, *32*, 84–87.
36. Bannai, A.; Ukawa, S.; Tamakoshi, A. Long working hours and sleep problems among public junior high school teachers in Japan. *J. Occup. Health* **2015**, *57*, 446–475.
37. Cho, S.S.; Ki, M.; Kim, K.H.; Ju, Y.S.; Paek, D.Y.; Lee, W.Y. Working hours and self-rated health over 7 years: Gender differences in a Korean longitudinal study. *BMC Public Health* **2015**, *15*, 1287.
38. Hino, A.; Inoue, A.; Kawakami, N.; Tsuno, K.; Tomioka, K.; Nakanishi, M., Mafune, K.; Hiro, H. Buffering effects of job resources on the association of overtime work hours with psychological distress in Japanese white-collar workers. *Int. Arch. Occup. Environ. Health* **2015**, *88*, 631–640.

39. Kim, B.H.; Lee, H.E. The association between working hours and sleep disturbances according to occupation and gender. *Chronobiol. Int.* **2015**, *32*, 1109–1114.
40. Kim, W.; Park, E.C.; Lee, T.H.; Kim, T.H. Effect of working hours and precarious employment on depressive symptoms in South Korean employees: A longitudinal study. *Occup. Environ. Med.* **2016**, *73*, 816–822.
41. Yoon, C.G.; Kang, M.Y.; Bae, K.J.; Yoon, J.H. Do working hours and type of work affect obesity in South Korean female workers? Analysis of the Korean community health survey. *J. Women's Health* **2016**, *25*, 2.
42. Lee, K.; Suh, C.; Kim, J.E.; Park, J.O. The impact of long working hours on psychosocial stress response among white-collar workers. *Ind. Health* **2017**, *55*, 46–53.
43. Ma, Y.; Wang, Y.J.; Chen, B.R.; Shi, H.J.; Wang, H.; Khurwolah, M.R.; Li, Y.F.; Xie, Z.Y.; Yang, Y.; Wang, L.S. Study on association of working hours and occupational physical activity with the occurrence of coronary heart disease in a Chinese population. *PLoS One* **2017**, *12*, e0185598.
44. Nakata, A. Long working hours, job satisfaction, and depressive symptoms: A community-based cross-sectional study among Japanese employees in small- and medium-scale businesses *Oncotarget* **2017**, 8, 53041–53052.
45. Cho, S.S.; Ju, Y.S.; Paek, D.; Kim, H.; Jung-Choi, K. The combined effect of long working hours and low job control on self-rated health: An interaction analysis. *J. Occup. Environ. Med.* **2018**, *60*, 475–480.
46. Ogawa, R.; Seo, E.; Maeno, T.; Ito, M.; Sanuki, M.; Maeno, T. The relationship between long working hours and depression among first-year residents in Japan. *BMC Med. Educ.* **2018**, *18*, 50.
